# Effects of Iodine Intake and Nutraceuticals in Thyroidology: Update and Prospects

**DOI:** 10.3390/nu12051491

**Published:** 2020-05-20

**Authors:** Daniela Bonofiglio, Stefania Catalano

**Affiliations:** 1 Department of Pharmacy, Health and Nutritional Sciences, University of Calabria, 87036 Rende (CS), Italy; 2 Centro Sanitario, University of Calabria, 87036 Rende (CS), Italy

Iodine is a microelement that is naturally present in some foods, added to others, and available as a dietary supplement. Iodine from the diet is converted into the iodide ion before it is absorbed throughout the gastrointestinal tract [1,2]. When iodide enters the circulation, the thyroid gland selectively concentrates it in the appropriate amounts required for thyroid hormone synthesis, and most of the remaining amount is excreted in the urine [3]. The iodine-replete healthy adult has about 15–20 mg of iodine, 70–80% of which is contained in the thyroid [4]. The estimated average requirement of iodine can be extrapolated from a median urinary iodine concentration of 100 μg/L, which corresponds roughly to 150 μg daily iodine intake [5]. Median urinary iodine concentrations of 100–199 μg/L in children and adults, 150–249 μg/L in pregnant women and >100 μg/L in lactating women indicate iodine intakes are adequate [6,7]. Values lower than 100 μg/L in children and non-pregnant adults indicate insufficient iodine intake, although iodine deficiency is not classified as severe until urinary iodine levels are lower than 20 μg/L. Seaweed (kelp, nori, kombu, and wakame) is one of the best food sources of iodine, although it is highly variable in its content [8]. Other dietary sources of iodine are seafood, dairy products (partly due to the use of iodine feed supplements and iodophor sanitizing agents in the dairy industry), milk, green beans and eggs [9]. Iodine is also present in human breast milk, in infant formulas and in many multivitamin/mineral supplements, which contain iodine in the forms of potassium iodide or sodium iodide [7,10,11]. Over the last years, there has been a growing interest in some nutraceuticals, including selenium, carnitine, myo-inositol, flavonoids, omega-3 polyunsaturated fatty acids, resveratrol and vitamins, for their potential role in thyroid function [12,13,14]. Nutraceuticals could represent an opportunity in the prevention and treatment of some thyroid diseases, even though their effective action and high safety level should need to be supported by large clinical outcome trials. However, iodine remains the essential element for the thyroid gland being the key component of the thyroid hormones thyroxine (T4) and triiodothyronine (T3), which regulate a wide variety of physiological processes, such as protein synthesis and enzymatic activity, and are critical determinants of metabolic activity. They are also required for proper skeletal and central nervous system development in fetuses and infants [15].

Thyroid function is primarily regulated by the thyroid-stimulating hormone (TSH), secreted by the pituitary gland, which increases thyroidal uptake of iodine and stimulates the synthesis and release of T3 and T4. In the presence of inadequate iodine intake, TSH levels remain elevated, leading to goiter, an enlargement of the thyroid gland that reflects the body’s attempt to trap more iodine from the circulation and produce thyroid hormones.

Iodine deficiency impairs thyroid hormone production and has many adverse effects during the course of the life, collectively termed the iodine deficiency disorders (IDDs), which depend on its severity and the age of the affected subjects. Although goiter is the classic sign of iodine deficiency, and can take place at any age, the most serious adverse effect of iodine deficiency is damage to the fetus during pregnancy, since the absence or inadequate level of thyroid hormones cause significant clinical manifestations such as increased risk of stillbirths, abortions, perinatal mortality, congenital abnormalities, cretinism, impaired growth [9,16]. Nowadays, IDDs are still a public health problem in most countries, including industrialized and developing regions of the world, in which all groups of people are affected, even though pregnant women are the most susceptible group to insufficient iodine intake [17,18,19,20].

Over the last few decades, intensive efforts have been made by the governments of IDD-affected countries to implement and control salt iodization program, since the most cost-effective strategy for IDD is universal salt iodization with the recommended iodine concentration of 20–40 mg iodine per kg salt [5]. Despite a significant improvement in iodine nutrition being observed over the years, some countries still remain at risk of deficiency, implying that further strategies should be designed to achieve iodine sufficiency worldwide.

In this issue, we provide an update on the iodine status of the general population in different countries of the world, including Moldovia [21], Korea [22], the United States [23,24], Sri Lanka [25], Finland [26] and Italy [27,28], with a special focus on newborns [24] and pregnant women [24,26], which are the most vulnerable categories. Importantly, we have invited an international panel of endocrinologists to review the literature and to comment upon the effects of the most common nutraceuticals on thyroid function, based on the most recent in vitro and in vivo [29,30], as well as human, studies [31]. We believe that this collection represents a useful summary of iodine intake on human health, and provides a critical opinion on the benefits of some popular nutraceuticals that may play a role in clinical thyroidology.
